# ART restorations for occluso-proximal cavities in primary molars: a two-year survival and cost analysis of an RCT comparing two GIC brands

**DOI:** 10.1590/1678-7757-2022-0148

**Published:** 2022-11-04

**Authors:** Isabel Cristina OLEGÁRIO, Anna Luiza de Brito Pacheco FURLAN, Caroline Mariano LAUX, Daniela HESSE, Clarissa Calil BONIFÁCIO, José Carlos Pettorossi IMPARATO, Daniela Prócida RAGGIO

**Affiliations:** 1 Dublin Dental University Hospital Department of Public & Child Dental Health Trinity College Dublin Ireland Dublin Dental University Hospital, Department of Public & Child Dental Health, Trinity College, Dublin, Ireland.; 2 Faculdade São Leopoldo Mandic Faculdade de Odontologia Centro de Pesquisa Odontológica Campinas SP Brasil Faculdade São Leopoldo Mandic, Faculdade de Odontologia, Centro de Pesquisa Odontológica, Campinas, SP, Brasil.; 3 Universidade de São Paulo Faculdade de Odontologia Departamento de Ortodontia e Odontopediatria São Paulo SP Brasil Universidade de São Paulo, Faculdade de Odontologia, Departamento de Ortodontia e Odontopediatria, São Paulo, SP, Brasil.; 4 Academic Centre for Dentistry Amsterdam Department of Pediatric Dentistry Amsterdam Netherlands Academic Centre for Dentistry Amsterdam (ACTA), Department of Pediatric Dentistry, Amsterdam, the Netherlands.; 5 Clinical Dental Researcher Cardiff University Cardiff United Kingdom Clinical Dental Researcher in Cardiff University, Cardiff, United Kingdom.

**Keywords:** Atraumatic Restorative Treatment, Glass ionomer cement, Clinical trial, Cost-effectiveness, Restoration survival percentage, Primary teeth, Pediatric dentistry

## Abstract

There are many glass ionomer cements available on the Brazilian market for Atraumatic Restorative Treatment (ART), however, there is still a gap in the literature regarding their cost-effectiveness.

**Objectives:**

To evaluate the influence of restorative materials (Ketac Molar, 3M ESPE; and Vitro Molar, Nova DFL) in the two-year survival rate and cost-effectiveness of occluso-proximal ART restorations in primary molars.

**Methodology:**

A total of 117 children (aged four to eight years) with at least one occluso-proximal carious lesion in primary molars were selected and randomly divided in treatment groups (KM or VM) in this parallel randomized controlled trial. Treatments followed ART premises and were conducted in public schools by trained operators in Barueri, Brazil. A trained, calibrated, and blinded examiner performed the evaluations after two, six, 12, and 24 months (k=0.92). Kaplan-Meier survival analysis was used to estimate restoration survival and Cox regression was used to test the association with clinical factors (α=5%). For cost analysis, material and professional costs were considered. Monte Carlo analysis was used to generate a cost-effectiveness plane and bootstrapping was used to compare material costs over the years.

**Results:**

The overall survival rate was 36.9% after two years (48.6% for KM and 25.4% for VM). Restorations with VM failed more than those with KM (HR=1.70; 95% CI=1.06–2.73; p=0.027). VM presented lower initial cost, but no difference was observed between groups considering the two-year incremental cost.

**Conclusion:**

After a two-year evaluation, KM proved to be a better option than VM for occluso-proximal ART restorations in primary molars. ClinicalTrials.gov: NCT02267720

## Introduction

Restorative treatment for multi-surface cavitated carious lesions is challenging in Pediatric Dentistry.^1^ Although evidence suggests that the Hall technique is the best alternative to treat primary teeth, the costs and availability of crowns could be barriers to their implementation in Brazil and Latin American countries. When comparing tooth-colored adhesive options, the effectiveness of restoration of the glass ionomer cement (GIC) and Atraumatic Restorative Treatment (ART) techniques has been evaluated by several randomized clinical trials and systematic reviews.^2 , 3^ Although ART emerged in the mid-1980s as an alternative for dental care in underserved communities, it is now considered a patient-friendly approach under field conditions or in clinical setting.^4^

High-viscosity GIC is the material of choice for ART restorations and a large variety of GIC brands are available in the dental market. To guide stakeholders regarding the GIC material, a recent consensus^5^determined thresholds for GIC restorative indications based on compressive strength, microhardness, acid erosion, and fluoride release. However, there is still a need for clinical trials to help decision-makers translate the consensus findings into long-term clinical survival and cost-effectiveness results.

Ketac Molar Easy Mix (3M ESPE, Seefeld, Germany) is considered one of the most effective materials for ART restorations, as it shows proper clinical performance and longevity.^6 - 11^ However, more affordable options in the Brazilian market are already included in public health services. Vitro Molar^12 - 17^ (Nova DFL) is one of those, which is considered a low-cost option with favorable mechanical characteristics and fluoride release.^6^ However, more clinical trials are needed to evaluate the long-term survival and cost-effectiveness of GIC materials.^6 , 7^

Economic evaluation, such as cost-effectiveness analyses, support clinical decision-making.^18^ Evaluating efficacy/effectiveness (treatment survival) and treatment cost (baseline and incremental cost evaluation over time) can provide the information needed to structure treatment pathways based on the perspective analyzed. Therefore, this randomized clinical trial aimed to evaluate the influence of restorative materials (Ketac Molar and Vitro Molar) on the two-year survival rate of occluso-proximal ART restorations. As a secondary outcome, cost-effectiveness analyses were performed, considering baseline and incremental prospected costs between groups at the 6-, 12-, and 24-month evaluation.

## Methodology

This study followed the Consolidated Standards of Reporting Trials (CONSORT), and used the Consolidated Health Economic Evaluation Reporting Standards (CHEERS) to report economic data (checklists available in Supplementary Materials 1 and 2, respectively).

### Study design

This two-arm parallel single-blinded randomized clinical trial was registered on the Clinical Trials website under registration no. NCT02267720 and approved by the Research Ethics Committee of the School of Dentistry, University of São Paulo (#569.112).

One-year clinical results and detailed protocol description, including sample size estimation, have already been published.^7^ The selection of participants and all treatments were performed in public schools in Barueri, São Paulo, Brazil.

### Deviations from the protocol

Sample size estimation was based on the primary outcome (survival of occluso-proximal ART restorations), as described in ClinicalTrials.gov. Although cost-effectiveness analysis was previously registered as another primary outcome, it was the secondary outcome of this study.

### Eligibility criteria

Healthy children aged four to eight years with at least one primary molar with an occluso-proximal carious lesion were the inclusion criteria. Clinical pulp exposure, tooth mobility, swelling, fistula near the tooth, or a lesion inaccessible to hand instruments were the exclusion criteria.

Only one tooth per child was included and if the child had more than one tooth that met the inclusion criteria, a simple draw was performed to select which tooth would be included. If the child had other teeth that required restorative treatments and fissure sealants, the study team also performed these treatments. Only treatments that could not be performed outside dental facilities (such as tooth extraction and root canal treatment) were treated by the nearest public dental clinic.

### Operators

Two undergraduate students trained according to the ART approach performed all restorations. The students underwent an one-week laboratory and clinical training at the University of São Paulo under the supervision of an expert (DPR). Moreover, a trained dental assistant from the public health system was responsible for mixing GICs according to the manufacturers’ instructions. The dental assistant was properly trained by an ART expert on how to dose and mix the two GICs according to the manufactures’ instructions.

The students were working together, so, there was enough time for the assistant to mix GICs. She was trained to dose and mix materials before the registration of the first participant enrolment.

### Study setting

This study was conducted in public schools in Barueri, São Paulo, Brazil. All treatments and evaluations were performed on school desks, in empty classrooms, with no access to dental equipment, such as rotary instruments, 3-in-1 air-water syringe, and suction devices. To improve the visibility of the work area, operators used front lights.

### Randomization, blinding, and allocation concealment

The randomization process was performed by the website randomization.com and designed in blocks of different sizes (four, six, and eight). After selective caries removal and cavity volume measurements, a dental assistant opened sealed, sequentially numbered, and opaque envelopes. Children were randomly assigned to the KM (Ketac Molar Easy Mix, 3M ESPE) or VM (Vitro Molar, NOVA DFL) groups. Although the materials presented similar colors, their thicknesses were not identical; therefore, operators could not be blinded.

### Interventions

All children were treated on school desks in empty classrooms. All restorations were performed according to the protocol proposed by Frencken and Holmgren^19^ (1999).

Selective caries removal was performed using hand instruments under cotton roll isolation. After caries removal, the cavity volume was measured using a periodontal probe. Randomization was performed at this stage by a dental assistant responsible for material handling. The cavity was conditioned with polyacrylic acid using a microbrush for 10 seconds, rinsed with wet cotton pellets (3), and dried with cotton pellets (3). A metal matrix and wooden wedge were positioned for restoration. Cavities were restored using two GICs: KM (3M ESPE, Seefeld, Germany) and VM (Nova DFL). According to the manufacturers’ instructions, both GICs were dosed, hand-mixed (powder/liquid ratio 1:1) and inserted into the cavity. A thin layer of petroleum jelly was rubbed over the index finger of the operator and the material was held under pressure for 20 seconds. After the initial setting of the material (three minutes and 30 seconds for KM and four minutes for VM), the matrix and wedge were removed and the occlusion was checked using an articulating paper. Excess material was removed using dental excavators and a new layer of petroleum jelly was applied.

All information related to participants (sex, age, caries experience, DMFT/dmft) and the clinical characteristics of their occluso-proximal carious lesion (surface: mesial/distal; jaw: upper/lower; molar: first or second primary molar; and cavity volume) were collected by the operators.

A research assistant recorded the time of each procedure, from when children were seated until the restoration was finished, and wrote down all materials used during the procedure (e.g., cotton rolls, GIC doses, dental floss, among others).

A single-blinded independent examiner evaluated the restorations at school after two, six, 12, and 24 months. The evaluation was performed using the criteria described by Roeleveld, et al.^20^ (2006). Scores 0 or 10 were considered successful whereas scores 11, 12, 13, 20, 21, 30, 40, or 50 were considered restoration failures. The other scores (60, 70, and 90) were censored for survival analysis.

### Analysis

Data analysis was performed using the Stata SE 17.0 software. The chi-square test was performed to evaluate the equivalence of categorical variables. After two years, the survival rate of restorations was assessed using Kaplan-Meier survival analysis and the log-rank test. To evaluate the association between outcomes and participant-related variables, Cox regression models were created. Only the variables that reached p<0.20 in the univariate analysis were included in the adjusted model.

For cost analysis, two components were considered: professional and material costs, based on a previous study.^17^ All costs were converted from Brazilian reais (R$) to US dollars (US$) using purchasing power parities (PPP), currency values from 2020 (US$1=R$2.31).^21^ Brazilian Federal Law No. 3991/61 was considered to determine the professional cost. The average of different dental material supplies was used to determine the material cost. Depending on the success or failure of the restoration, a prospective estimation was made for the incremental cost. For score 30 (no restoration, bulk fracture, or partial loss of the restoration), the restoration replacement was estimated to cost the same as the baseline (total cost=2×baseline cost), while a restoration repair (scores 11, 12, 13, 20, or 21) was estimated to cost 50% of the baseline (total cost=1.5×baseline cost). The incremental cost for a successful restoration (scores 0 and 11) was zero (total cost=baseline cost). Only one case of failure per restoration was considered.^17^

The initial (baseline) and incremental prospected costs at the two-year follow-up were calculated and compared using bootstrapping regression analysis (1,000 replications).

To present the incremental cost and survival of the VM group in comparison with the KM group, the Bayesian approach was used to create a cost-effectiveness plane using Monte Carlo analysis (10,000 simulations), considering the differences in the material cost (delta cost) and survival time (delta time). XLSTAT 2020 (Addinsoft SARL, Paris, France) was used for this analysis. The level of significance was 5% for all analyses.

## Results

A total of 548 children were screened in 19 public schools in Barueri, São Paulo, Brazil, of which 117 children aged four to eight years met the inclusion criteria and were selected for the study. Among these children, 58 were assigned to the VM group and 59 to the KM group. The main reasons for exclusion were the absence of primary molar with an occluso-proximal carious lesion eligible for the study, clinical pulp exposure, tooth mobility, swelling, or fistula near the tooth (n=38).

**Figure 1:**
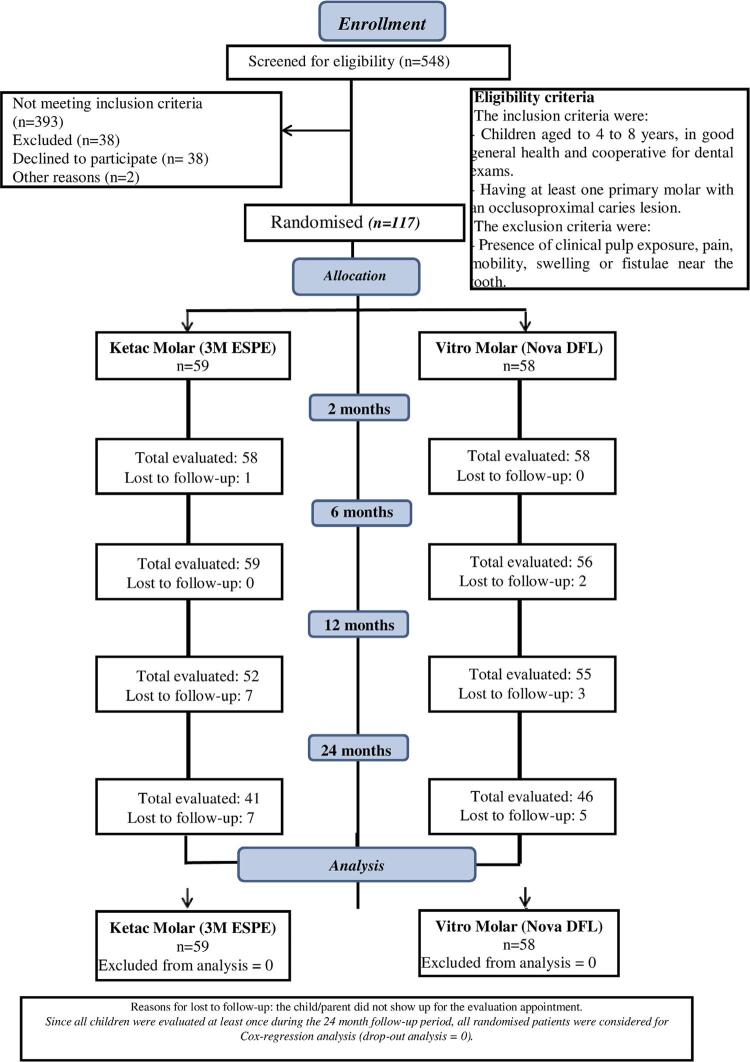
CONSORT flow diagram

Participants were recruited in November 2014 and restorative treatments were performed from February to March 2015. The follow-up evaluation started after restorations and was completed after 24 months. Figure 1 presents the flowchart proposed by CONSORT for clinical trials with detailed information regarding the number of participants in all follow-up periods.

The intra-examiner weighted kappa value was 0.92. All children were evaluated at least once (at two, six, 12, or 24 months) during the follow-up period. Therefore, no child was excluded from the survival analysis.

Table 1 shows the distribution of baseline demographic and clinical characteristics (sex, age, caries experience, surface, jaw, molar, and cavity volume) for each group. All variables were equally distributed among the studied groups (p>0.05).

**Table 1:** Distribution of baseline variables for each group

	Ketac Molar	Vitro Molar	Remained	Dropped out
**TOTAL N (%)**	59 (50.43)	58 (49.57)	107 (91.45)	10 (8.55)
Categorical variables – N (%)				
**Sex**				
Men	29 (46.77)	33 (53.23)	58 (93.55)	4 (6.45)
Women	30 (54.55)	25 (45.45)	49 (89.09)	6 (10.91)
**Age**				
3–5 years old	41 (52.56)	37 (47.44)	76 (97.44)	2 (2.56)
>5 years old	18 (46.15)	21 (53.85)	31 (79.49)	8 (20.51)
**Caries experience (DMFT)**				
0–3	20 (40.82)	29 (59.18)	45 (91.84)	4 (8.16)
>3	39 (57.35)	29 (42.65)	62 (91.18)	6 (8.82)
**Surface**				
Occluso-mesial	14 (45.16)	17 (54.84)	27 (87.10)	4 (12.90)
Occluso-distal	45 (52.33)	41 (47.67)	80 (93.02)	6 (6.98)
**Jaw**				
Upper	26 (50.00)	26(50.00)	45 (86.54)	7 (13.46)
Lower	33 (50.77)	32 (49.23)	62 (95.38)	3 (4.62)
**Molar**				
First primary molar	52 (53.61)	45 (46.39)	91 (93.81)	6 (6.19)
Second primary molar	7 (35.00)	13 (65.00)	16 (80.00)	4 (20.00)
**Volume**				
0–9.99mm3	46 (48.42)	49 (51.58)	88 (92.63)	7 (7.37)
≥10mm3	13 (59.09)	9 (40.91)	19 (86.36)	3 (13.64)

DMFT = number of decayed, missing due to caries, and filled primary teeth.

* Seven children who dropped out were from the KM group and three were from the VM group (p=0.322, by the Fisher exact test)

**Figure 2:**
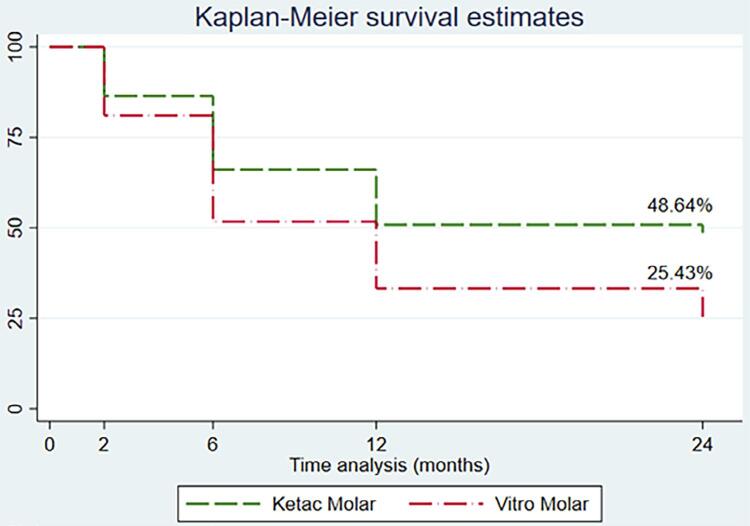
Kaplan-Meier survival analysis

After 24 months, the overall survival rate of restorations was 36.97%. The survival rate was 48.64% for the KM group and 25.43% for the VM group. The main reason for restoration failure at 24 months was bulk fracture (79.17%; n=57). Figure 2 presents the Kaplan-Meier survival analysis, showing a difference between the survival curves of the studied groups (log-rank=0.019).

Table 2 shows the Cox regression analysis between independent variables and restoration failure. The GIC material influenced restoration survival (HR=1.70; 95%CI=1.06–2.73; p=0.027). No other tested variable (sex, age, caries experience, jaw, or cavity volume) influenced the survival rate of restorations.

**Table 2:** Univariate and adjusted Cox Regression analysis for restoration failure between independent variables

Variable	Two-year Survival rate %	95%CI	HR Univariated† 95%CI ‡	p-value	HR Ajusted† 95%CI ‡	Two-tailed p-value
**Group**						
Ketac Molar (ref)	48.64	35.28–60.74	1.62	0.043*	1.7	0.027*
Vitro Molar	25.43	14.81–37.46	1.01–2.59		1.06–2.73	
**Sex**						
Men (ref)	39.13	26.84–51.21	1.13	0.595	-	-
Women	34.52	21.91–47.47	0.71–1.79			
**Age**						
3–5 years old (ref)	35.56	25.08–46.16	0.9	0.692	-	-
>5 years old	40.13	23.79–55.96	0.54–1.49			
**Caries experience (DMFT)**						
0–3 (ref)	31.4	18.18–44.79	0.8	0.35	-	-
>3	41.02	28.97–52.68	0.50–1.28			
**Surface**						
Occluso-mesial (ref)	56.53	37.02–72.08	1.83	0.047*	1.57	
Occluso-distal	30.05	20-49–40.18	1.01–3.35		0.68–3.61	
**Jaw**						
Upper (ref)	43.88	29.64–57.23	1.28	0.307	-	-
Lower	31.65	20.67–43.19	0.79–2.05			
**Molar**						
First primary molar (ref)	33.05	23.75–42.64	0.55	0.115	0.72	0.538
Second primary molar	57.69	32.65–76.31	0.26–1.15		0.26–2.01	
**Volume**						
0–9.99mm3	36.31	26.51–46.16	0.94	0.849	-	-
≥10mm3	40.91	20.85–60.07	0.52–1.72			
**TOTAL**	36.97	28.06–45.88				

HR: hazard ratio ; CI: confidence interval; * p<0.05 - 95%CI

Variables that reached p<0.20 in the univariated analysis were brought to the adjusted model (Log-likelihood=−316.99; p=0.022).

**Figure 3:**
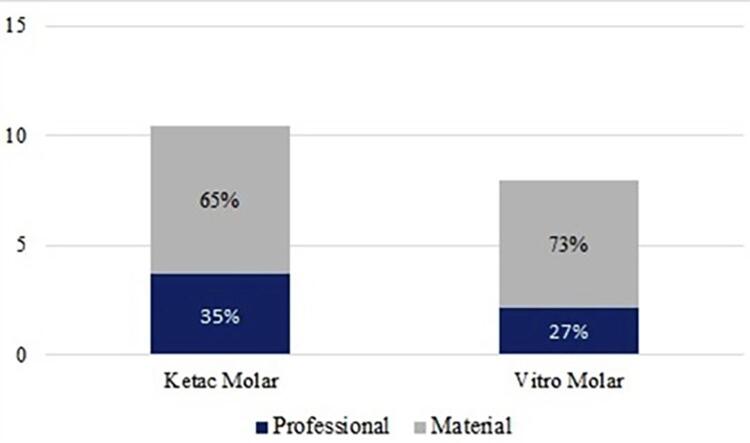
Distribution of material and professional costs

The mean±SD spent in minutes for restoration was 12.84±3.46 (13.84±3.25 for KM and 11.82±3.39 for VM). The total cost of restorations was estimated based on the material and professional expenses. The mean±SD total cost was US$9.17±2.33 and more than 68% of the total cost was due to professional expenses (mean±SD=US$6.2±1.68 per restoration). Figure 3 shows the distribution of material and professional costs. Table 3 presents the material cost analysis over time using bootstrapping regression analysis. The total cost of performing restorations with KM was US$10.39±2.00 while restorations with VM cost 24% less (US$7.92±1.95; p<0.001). However, considering the cost after two years, no difference was found between groups, showing that the restoration cost between materials is equivalent over time (p=0.075) due to different repair needs and prospected repair costs.

**Table 3:** Material cost evaluation over time using bootstrapping regression analysis (1,000 replications). All costs were converted from Brazilian reais (R$) to US dollars (US$); conversion rate: US$1=R$2.31

	Prospected mean US dollar (SD)	Coefficient (bootstrapping SE)	p-value	95%CI
**Baseline total cost**				
Ketac Molar (ref)	10.39 (2.00)			
Vitro Molar	7.92 (1.95)	−2.46 (0.36)	<0.001*	−3.18 to −1.75
**Six-month total cost**				
Ketac Molar (ref)	14.08 (5.94)			
Vitro Molar	11.96 (5.39)	−2.11 (1.05)	0.045*	−4.18 to −0.04
**One-year total cost**				
Ketac Molar (ref)	15.71 (6.45)			
Vitro Molar	13.30 (5.63)	−2.40 (1.13)	0.034*	−4.63 to −0.18
**Two-year total cost**				
Ketac Molar (ref)	15.71 (6.45)			
Vitro Molar	13.75 (5.34)	−1.95 (1.10)	0,075	−4.11 to 0.20

**Figure 4:**
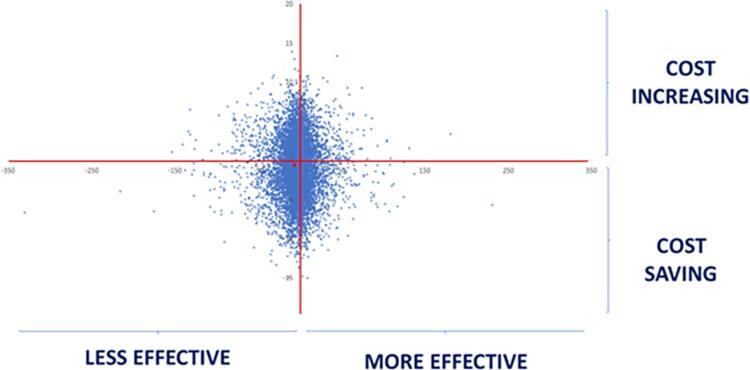
Cost-effectiveness plan for Vitro Molar in comparison with Ketac Molar

Figure 4 presents the cost-effectiveness plan. The effectiveness of VM was lower in comparison with KM. The distribution of dots is slightly displaced to the left lower quadrant, showing that the cost of VM was similar to KM, but its effectiveness was lower after two years.

### Harms

No harm was found in this study.

## Discussion

This study evaluated the survival rate and cost-effectiveness of occluso-proximal ART restorations performed in public schools using two brands of glass ionomer cement. The overall survival rate after two years (36.9%) found in this study was similar to that found by other authors when restoring occluso-proximal cavities and delivered by final-year dental students. Kemoli, et al.^11^ (2009) found an overall survival rate of 30.8% when comparing two ART GIC brands (Ketac Molar and Fuji IX) while Bonifacio, et al.^22^ (2013) found a survival rate of 27% after three years when comparing three different brands. For Da Franca, et al.^23^ (2011), the survival rate was 27.6% for restorations with KM after 24 months. Although this survival rate is lower than other minimal intervention strategies, such as the Hall technique,^24^ it is comparable to other tooth-colored restorations, such as composite resin and compomer.^2^ Although it has been proven that the operator performance can directly influence the longevity of the restoration,^3 , 25^ in our study, operators (fifth-year students) received adequate training and calibration from an expert in the field, which may decrease the risk of errors in procedures. Moreover, the technique used was less sensitive than other comparable restorative techniques (e.g., resin-based materials). Jiang, et al.^3^ (2020) confirms that our survival rate results are in line with the available evidence, ranging from 35% (26-44%) two years after the occluso-proximal restorations performed by undergraduate students. Restorations performed by dentists had a small increase in the survival rate (around 50%);^3^ this result is comparable with the rate of our KM group (48.6%), in which restorations was performed by dental students. Although ART is not a very sensitive technique, sufficient and proper training are necessary for both dentists and undergraduate students to perform successful restorations.

In our view, training and calibrating students to perform ART restorations can also help stakeholders to evaluate potential results when non-dentists (or non-experienced professionals) deliver restorative dental care. This change in the role of professionals can be called “task shifting.” The World Health Organization (WHO) made recent recommendations around task shifting.^26^ The operators in this study were ACTA (Academic Centre for Dentistry Amsterdam) students and we had an agreement for the mobility of students between The Netherlands and Brazil as part of their final-year project.

The major failures were bulk fractures (n=57) or partial restoration loss requiring repair (score 30). One feature of occluso-proximal dentine carious lesions is the loss of enamel in the gingival margin, which results in a subgingival extension of the restoration. Consequently, it compromises the adhesion of GIC, which depends mainly on interactions with the tooth structure (enamel and dentin). Along with residual caries, these cervical gaps represent the main factor influencing the survival of these restorations.^20^

Another factor contributing to the failure of ART restorations is the difficulty in performing adequate caries removal in small cavities using hand instruments.^8^ A recently published systematic review showed that inadequate caries removal could influence the survival of ART restorations.^3^ Our results showed that the main factor associated with restoration survival was the type of material. In contrast, no other variable, such as sex, jaw, cavity volume, tooth type, and caries experience, significantly influenced the survival rate.

Although restorations with VM initially had a lower cost (US$7.92) than those with KM (US$10.39), when considering the simulation of the two-year cost, no differences were found between materials regarding material and professional cost. Consequently, KM is a better material to restore occluso-proximal cavities, since it has a higher survival rate and does not require as many repairs as VM. However, we simulated the repair cost, which can lead to an underestimation of treatment costs. Only one repair was considered in the cost analysis, representing one of the limitations of this study. Another potential limitation is GIC dosage and handling. As an RCT, all operators were trained and performed the procedures correctly. We did not anticipate the same results in an effectiveness trial (or in a real-world setting).

Therefore, cost-effectiveness is crucial when choosing the ideal treatment/material for each case. Clinical trials that include cost analysis, such as this study, have an essential and decisive role in the decision-making process, especially regarding technique and material choice. When evaluating the cost results, VM was the cheapest option at baseline and in the one-year follow-up ( Table 3 ). However, no differences were found in the final cost due to the lower survival rate and increased need for replacement at the two-year follow-up (p=0.075). Moreover, the cost-effectiveness plan corroborates this finding with an equal distribution of costs. In turn, Figure 4 shows that VM was considered “less effective” when compared with KM.

As a secondary outcome result, we observed that the mean time spent in minutes per restoration was similar (13.84 minutes for KM and 11.82 for VM). Similar results were found by Da Mata, et al.^27^ (2014), who reported an average ART restoration time of 13 minutes with older adults.

Our results should be considered in the decision-making process when choosing GIC materials for ART restorations in primary molars. The perspective used in the current analysis was the Brazilian public health system and all costs were measured based on the values of material and professional costs locally available.^17^ Therefore, the cost of restorative treatments may differ between countries and the cost results presented in this study cannot be extrapolated to different settings.

## Conclusion

After a two-year evaluation, KM proved to be a better option than VM for occluso-proximal ART restorations in primary molars.
